# Diversity in Natural Transformation Frequencies and Regulation across *Vibrio* Species

**DOI:** 10.1128/mBio.02788-19

**Published:** 2019-12-17

**Authors:** Chelsea A. Simpson, Ram Podicheti, Douglas B. Rusch, Ankur B. Dalia, Julia C. van Kessel

**Affiliations:** a Department of Biology, Indiana University, Bloomington, Indiana, USA; b Center for Genomics and Bioinformatics, Indiana University, Bloomington, Indiana, USA; University of Illinois at Chicago

**Keywords:** competence, natural transformation, vibrio, quorum sensing

## Abstract

In *Vibrio* species, chitin-induced natural transformation enables bacteria to take up DNA from the external environment and integrate it into their genome. Expression of the master competence regulator TfoX bypasses the need for chitin induction and drives expression of the genes required for competence in several *Vibrio* species. Here, we show that TfoX expression in Vibrio campbellii strains DS40M4 and NBRC 15631 enables high natural transformation frequencies. Conversely, transformation was not achieved in the model quorum-sensing strain *V. campbellii* BB120 (previously classified as Vibrio harveyi). Surprisingly, we find that quorum sensing is not required for transformation in *V. campbellii* DS40M4 or Vibrio parahaemolyticus in contrast to the established regulatory pathway in Vibrio cholerae in which quorum sensing is required to activate the competence regulator QstR. Similar to V. cholerae, expression of both QstR and TfoX is necessary for transformation in DS40M4. There is a wide disparity in transformation frequencies among even closely related *Vibrio* strains, with V. vulnificus having the lowest functional transformation frequency. Ectopic expression of both TfoX and QstR is sufficient to produce a significant increase in transformation frequency in Vibrio vulnificus. To explore differences in competence regulation, we used previously studied V. cholerae competence genes to inform a comparative genomics analysis coupled with transcriptomics. We find that transformation capability cannot necessarily be predicted by the level of gene conservation but rather correlates with competence gene expression following TfoX induction. Thus, we have uncovered notable species- and strain-level variations in the competence gene regulation pathway across the *Vibrio* genus.

## Abstract (precis)

Naturally transformable, or competent, bacteria are able to take up DNA from their environment, a key method of horizontal gene transfer for acquisition of new DNA sequences. Our research shows that *Vibrio* species that inhabit marine environments exhibit a wide diversity in natural transformation capability ranging from nontransformability to high transformation rates in which 10% of cells measurably incorporate new DNA. We show that the role of regulatory systems controlling the expression of competence genes (e.g., quorum sensing) differs throughout both the species and strain levels. We explore natural transformation capabilities of Vibrio campbellii species which have been thus far uncharacterized and find novel regulation of competence. Expression of two key transcription factors, TfoX and QstR, is necessary to stimulate high levels of transformation in Vibrio campbellii and recover low rates of transformation in Vibrio vulnificus.

## INTRODUCTION

Natural transformation is a process in which cellular physiology changes, thereby allowing bacteria to take up extracellular DNA from the environment, transport it across the cell envelope, and integrate it into their genome via homologous recombination. In some marine *Vibrio* species, competence is induced by growth on the chitinous exoskeletons of crustaceans (1, 2). This process has been best studied in Vibrio cholerae (reviewed in reference 2). Insoluble polysaccharide chitin is broken down by secreted extracellular chitinases (3). Soluble chitin oligosaccharides ultimately induce expression of the master competence regulator TfoX (4), which activates expression of numerous components of the competence machinery required to take up extracellular DNA. In addition to the chitin-sensing system, V. cholerae cells must also have a functional quorum-sensing system for natural transformation to be successful (1). Quorum sensing, the process of cell-cell communication, allows bacterial cells to respond to changes in population density and alter gene expression (5). The quorum-sensing systems in *Vibrio* species rely on detection of extracellular autoinducers (AIs) that are sensed by membrane-bound sensor kinases, which shuttle phosphate to or away from the core response regulator LuxO at low cell density or high cell density, respectively (5). At the end of this phosphorylation cascade at high cell density, the master transcription factor called LuxR is expressed, which controls expression of hundreds of genes (6). LuxR is the name of the master transcription factor in Vibrio campbellii (previously called Vibrio harveyi), whereas the homologs in other *Vibrio* species have different names: HapR (V. cholerae), SmcR (Vibrio vulnificus), and OpaR (Vibrio parahaemolyticus) (7). In V. cholerae, Δ*hapR* mutants are not competent, and this is due to HapR regulation of various competence genes, including *qstR* and *dns* (1, 8, 9, 30). HapR directly activates *qstR* (encoding a transcriptional regulator) and represses *dns* (encoding an extracellular DNase) (8). QstR subsequently activates downstream genes required for DNA uptake and integration, such as *comEA*, *comEC*, and *comM* (10). The requirement for HapR for competence can be circumvented if *qstR* and *tfoX* expression are induced and the *dns* gene is deleted (10). However, in wild-type V. cholerae, functional chitin-sensing and quorum-sensing systems are required for competence.

Growth on chitin is sufficient to induce competence in several *Vibrio* species, including V. cholerae, V. vulnificus, and V. parahaemolyticus (1, 11–14). Anecdotally, chitin-dependent natural transformation has not been observed in other species. In these cases, it may be that other environmental signals are required to induce competence or that laboratory conditions for chitin sensing are not sufficient. Further, multiple studies have identified different transformation capabilities among V. cholerae strains (15, 16). However, because the chitin-sensing system drives expression of the core competence regulator TfoX, overexpression of Tfox is sufficient to bypass the requirement for chitin and induce competence in V. cholerae, V. vulnificus, V. parahaemolyticus, and Vibrio natriegens (1, 12, 17–19).

The type strain of Vibrio campbellii, called BB120 (or ATCC BAA-1116), is a model system for studying quorum sensing in *Vibrio* species (20). This strain was historically called Vibrio harveyi until a recent comparative genomics analysis reclassified it as *V. campbellii* (21). However, while BB120 is highly studied, the competence of this strain and others in this species was undetermined. Here, we show that the DS40M4 and NBRC 15631 environmental isolates of *V. campbellii* are highly competent following TfoX overexpression, whereas the lab strain BB120 and another environmental isolate called HY01 are not able to undergo natural transformation via either chitin induction or TfoX overexpression. We compare multiple *Vibrio* species and show that wide variation in transformation frequencies exists between and within different species. Further, we uncover a variation in the regulatory pathway controlling competence gene expression: quorum sensing is not required for transformation in *V. campbellii* DS40M4 or V. parahaemolyticus but is required in *V. campbellii* NBRC 15631, V. cholerae, and V. vulnificus.

## RESULTS

### Induction of natural transformation via TfoX overexpression in *V. campbellii* DS40M4 and NBRC 15631 strains.

Previous studies of natural transformation in *Vibrio* species suggest that the structure of the signaling cascade that promotes natural transformation is likely to be conserved among *Vibrionaceae*. However, anecdotal reports and unpublished experiments have indicated that the *V. campbellii* type strain BB120 lacks the ability to undergo natural transformation. To formally test this, we assayed for transformation of plasmid DNA into BB120 using both chitin-dependent and -independent methods to induce competence (19, 22). For chitin-dependent transformations, cells are incubated with powdered chitin from shrimp shells and DNA and plated with antibiotic selection. For chitin-independent transformations, a previously published plasmid expressing V. cholerae *tfoX* via an IPTG-inducible promoter (pMMB67EH-tfoX-kanR [see Table S2 in the supplemental material]) is used to induce competence in cells. Due to the high conservation of TfoX across *Vibrio* species (Fig. S1) and the successful use of V. cholerae *tfoX* to induce competence in *V. natriegens*, we reasoned that the V. cholerae TfoX protein would be functional in *V. campbellii*. After competence is induced, the cells are incubated with DNA prior to plating for antibiotic selection. We did not recover antibiotic-resistant colonies from either method in BB120, whereas high transformation frequencies were obtained with the same plasmid DNA introduced into positive-control strains (*V. natriegens* for chitin-independent transformation [Fig. S2A]; V. cholerae for chitin-dependent transformation [data not shown]).

10.1128/mBio.02788-19.2 FIG S1 Alignment of TfoX homologs from *Vibrio* strains. Protein sequences were aligned using Clustal Omega [F. Madeira, Y. M. Park, J. Lee, N. Buso, et al., Nucleic Acids Res 47(W1):W636–W641, 2019, https://doi.org/10.1093/nar/gkz268], and the diagrams were generated using Jalview 2.0 (A. M. Waterhouse, J. B. Procter, D. M. Martin, M. Clamp, and G. J. Barton, Bioinformatics, 25:1189–1191, 2009, https://doi.org/10.1093/bioinformatics/btp033). Download FIG S1, PDF file, 0.6 MB. Copyright © 2019 Simpson et al. 2019 Simpson et al. This content is distributed under the terms of the Creative Commons Attribution 4.0 International license.

10.1128/mBio.02788-19.3 FIG S2 (A) Chitin-independent transformations of *V. campbellii* strains BB120, DS40M4, NBRC 15631, and HY01 with plasmid tDNA compared to positive-control strain *V. natriegens.* Strains contained either a plasmid expressing *tfoX* (pMMB67EH-tfoX-kanR for *V. campbellii* strains) or an empty vector control (pMMB67EH-kanR for *V. campbellii* strains or pMMB67EH for *V. natriegens*). Strains were transformed with 1 μg of plasmid tDNA conferring chloramphenicol resistance (pJV298). LOD, limit of detection. (B) Chitin-independent transformation efficiencies in DS40M4 and NBRC 15631 reactions with varied quantities of cell inoculations. Strains were transformed with 50 ng linear *luxO*::Spec^r^ tDNA. Averaged results from two technical replicates are shown for each condition. (C) Chitin-independent transformation efficiencies in DS40M4 and NBRC 15631 reactions with varied quantities of linear *luxO*::Spec^r^ tDNA. Averaged results from two technical replicates are shown for each condition. (D) Chitin-independent transformation efficiencies in DS40M4 and NBRC 15631 reactions with varied outgrowth times following addition of tDNA. Strains were transformed with 50 ng linear *luxO*::Spec^r^ tDNA. Averaged results from two technical replicates are shown for each condition. Download FIG S2, PDF file, 0.2 MB. Copyright © 2019 Simpson et al. 2019 Simpson et al. This content is distributed under the terms of the Creative Commons Attribution 4.0 International license.

10.1128/mBio.02788-19.6 TABLE S2 Plasmids used in this study. Download Table S2, DOCX file, 0.01 MB. Copyright © 2019 Simpson et al. 2019 Simpson et al. This content is distributed under the terms of the Creative Commons Attribution 4.0 International license.

**FIG 1:**
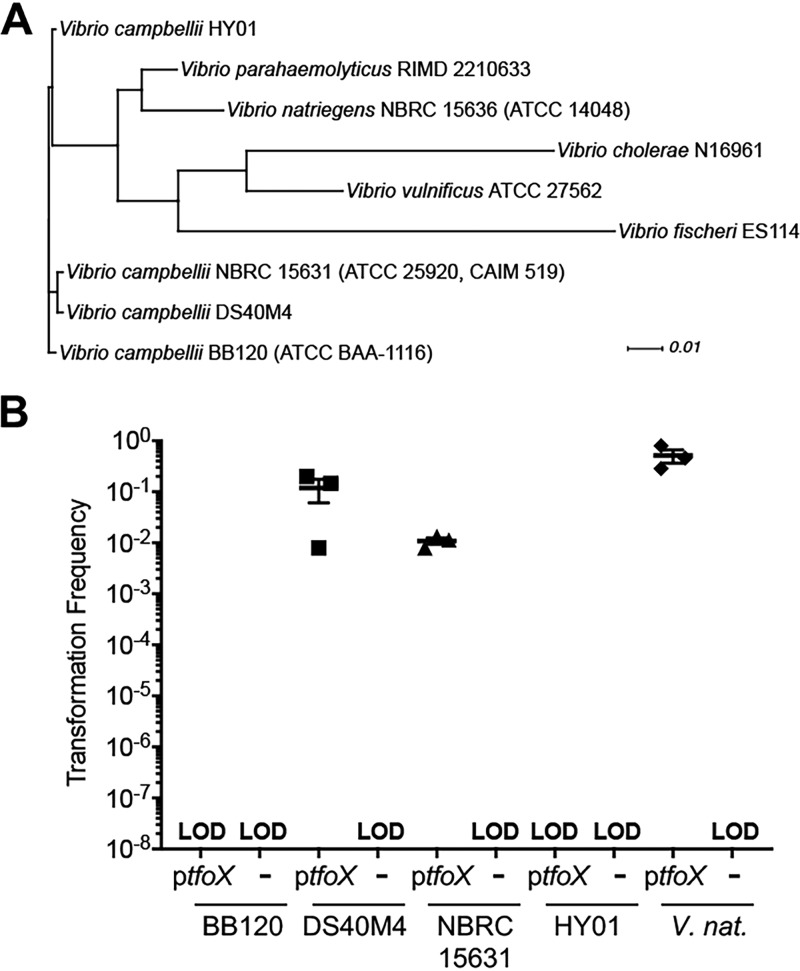
Natural transformation of *V. campbellii* strains via *tfoX* expression. (A) Phylogenetic tree of *Vibrio* strains based on comparison of amino acid sequences of 79 core conserved genes in the genomes shown. (B) Chitin-independent transformation of *V. campbellii* strains BB120, DS40M4, NBRC 15631, and HY01 compared to *V. natriegens*. Strains contained either a plasmid expressing *tfoX* (pMMB67EH-tfoX-kanR) or an empty vector control (pMMB67EH-kanR for *V. campbellii* strains or pMMB67EH-carbR for *V. natriegens*). Strains were transformed with 300 ng of linear *luxR*::Spec^r^ tDNA (for *V. campbellii*) or *dns*::Spec^r^ tDNA (for *V. natriegens*). LOD, limit of detection.

Because strains of V. cholerae exhibit differing natural transformation frequencies (15, 16), we hypothesized that the absence of competence in BB120 may not be indicative of competence in other *V. campbellii* isolates. We therefore assayed natural transformation in three *V. campbellii* environmental isolates with sequenced genomes: DS40M4, NBRC 15631, and HY01 (21, 23–26). DS40M4 was isolated from the Atlantic Ocean near the west coast of Africa (26), HY01 was isolated from a bioluminescent shrimp in Thailand (24), and NBRC 15631 (also called CAIM 519 and ATCC 25920) was isolated from seawater in Hawaii (21). These species cluster together in the Harveyi clade in the *Vibrio* genus, with DS40M4 and NBRC 15631 being the most closely related (Fig. 1A). Incubation on chitin was unable to induce cells to take up the plasmid DNA in any of the *V. campbellii* strains (data not shown). However, both the DS40M4 and NBRC 15631 isolates were able to undergo transformation of plasmid DNA using the chitin-independent method in which TfoX was ectopically expressed (Fig. S2A). Transformation was dependent on TfoX expression because strains containing the empty vector control did not produce transformants (Fig. S2A).

We next assayed transformation of linear DNA (referred to as transforming DNA [tDNA]). We generated linear recombination products targeting the *luxO* gene in each *V. campbellii* strain containing an antibiotic resistance cassette and 3 kbp of DNA homologous to the regions flanking *luxO* for each strain (Δ*luxO*::Spec^r^ substrates). High transformation frequencies were obtained for both DS40M4 and NBRC 15631 with their respective tDNAs that are dependent on the inducible expression of *tfoX* (Fig. 1B). We tested various parameters for transformation, including the number of cells in the reaction mixture, the amount of time that tDNA was incubated with cells before outgrowth, and the amount of tDNA added to the reaction mixture (Fig. S2B, C, and D). Using these optimized methods, we still did not observe any antibiotic-resistant colonies from transformations with either the BB120 strain or the HY01 strain (Fig. 1B). However, each transformation reaction mixture that is plated on nonselective medium yields 10^8^ viable colonies; thus, lack of tDNA transformation is not a result of poor growth or viability in this procedure. As a comparison, we routinely obtain >100,000 antibiotic-resistant colonies with DS40M4 per tDNA transformation. Thus, the absence of antibiotic-resistant colonies from a transformation is noted as limit of detection (LOD) when applicable in this work. We note that the DS40M4 strain is close to or as efficient as *V. natriegens* at transformation with linear tDNA (Fig. 1B), which has thus far been reported as the *Vibrio* strain displaying the highest transformation frequencies (19).

### Assessment of quorum-sensing phenotypes in *V. campbellii* strains.

**FIG 2:**
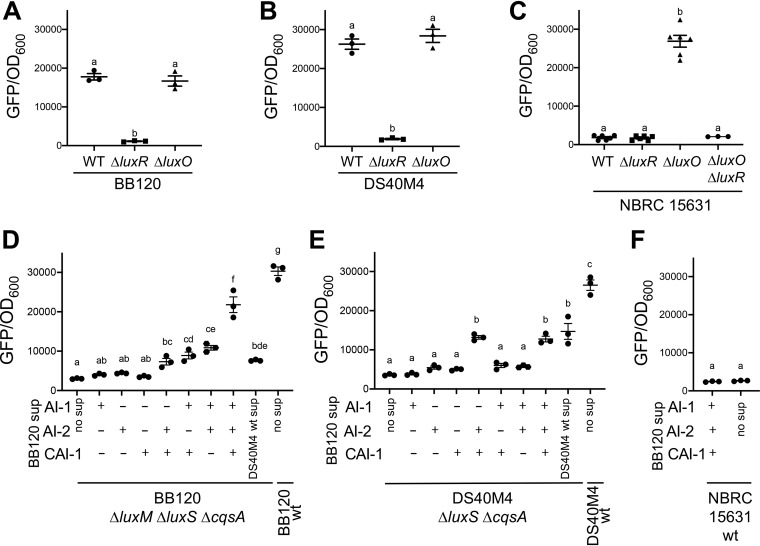
*V. campbellii* DS40M4 and NBRC 15631 encode functional LuxR and LuxO proteins. (A to C) The P*_luxCDABE_*-*gfp* reporter plasmid pCS019 was introduced into wild-type, Δ*luxR*, and Δ*luxO* strains of *V. campbellii* BB120 (A), DS40M4 (B), and NBRC 15631 (C), and the GFP fluorescence divided by OD_600_ was determined (GFP expression per cell). Different letters indicate significant differences (one-way analysis of variance [ANOVA] on log-transformed data, followed by Tukey’s multiple-comparison test, *P < *0.0001). (D to F) GFP expression per cell was determined as described above using the pCS019 reporter in BB120 Δ*luxM* Δ*luxS* Δ*cqsA* (CAS270) (D), DS40M4 Δ*luxS* Δ*cqsA* (CAS254) (E), and NBRC 15631 wild-type (F) strains in the presence or absence of supernatants (sup) from BB120-derived synthase mutant strains as indicated (TL184, AI-1; TL185, AI-2; TL16, CAI-1; JMH363, CAI-1, AI-2; KM816, CAI-1, AI-1; TL203, AI-1, AI-2; BB120, AI-1, AI-2, CAI-1). Different letters indicate significant differences (D and E, one-way analysis of variance [ANOVA] on log-transformed data, followed by Tukey’s multiple-comparison test, *P < *0.05; F, unpaired *t* test; *P < *0.05).

Because quorum sensing activates genes required for natural transformation in V. cholerae, we wanted to determine whether DS40M4 or NBRC 15631 contains functional quorum-sensing systems. Using chitin-independent natural transformation via *tfoX* induction, we generated Δ*luxO* and Δ*luxR* mutations in both the DS40M4 and NBRC 15631 strain backgrounds. In the model strain BB120, a Δ*luxO* mutant results in a constitutively expressed master quorum-sensing transcription factor, LuxR, producing high levels of bioluminescence and mimicking a high cell density phenotype (27). Conversely, a Δ*luxR* mutant of BB120 is unable to produce bioluminescence (28). We compared the phenotypes of wild-type, Δ*luxO*, and Δ*luxR* strains for BB120, DS40M4, and NBRC 15631 by assessing expression of the quorum-sensing bioluminescence genes, *luxCDABE*. We were unable to monitor bioluminescence because DS40M4 and NBRC 15631 do not contain all of the *luxCDABE* bioluminescence genes like BB120 (they carry only *luxB* homologs) and do not bioluminesce (data not shown). We therefore used a GFP reporter plasmid in which the BB120 bioluminescence promoter (P*_luxCDABE_*) is transcriptionally fused to *gfp*. In BB120, a Δ*luxR* strain exhibits a 16-fold decrease in GFP expression compared to the wild type, whereas a Δ*luxO* mutant exhibits similar GFP levels as the wild type (Fig. 2A). In the DS40M4 strains, we observed GFP expression similar to the analogous BB120 strains (Fig. 2B). We complemented the Δ*luxR* deletion strain in DS40M4 by integrating the wild-type allele into the chromosome under the control of its native promoter but in another locus, and the results showed that GFP expression is restored to wild-type levels (Fig. S3A).

10.1128/mBio.02788-19.4 FIG S3 (A) Complementation of Δ*luxR* and Δ*luxO* in DS40M4. The P*_luxCDABE_*-*gfp* reporter plasmid pCS019 was introduced into the strains of *V. campbellii*, and the GFP fluorescence divided by OD_600_ was determined. (B) Chitin-dependent transformations in each of the listed *Vibrio* species using Spec^r^ linear tDNAs (500 ng) targeting the *luxR* homolog in each strain. LOD, limit of detection. Download FIG S3, PDF file, 0.1 MB. Copyright © 2019 Simpson et al. 2019 Simpson et al. This content is distributed under the terms of the Creative Commons Attribution 4.0 International license.

The results with NBRC 15631 differ from BB120 and DS40M4. The Δ*luxO* mutant exhibits higher levels of GFP than the Δ*luxR* mutant, as predicted (Fig. 2C). Curiously, the wild-type NBRC 15631 strain consistently yields low levels of GFP, even though the cells reach similar densities as the wild-type strains of BB120 and DS40M4 (Fig. 2C). Importantly, deletion of *luxR* in the Δ*luxO* NBRC 15631 background eliminates GFP production, indicating that LuxR is required for GFP expression, and LuxR is epistatic to LuxO. To determine if NBRC 15631 responds to an autoinducer from another strain or species, we incubated NBRC 15631 cells in medium supplemented with cell-free supernatants from various *Vibrio* strains (including DS40M4). The GFP levels in the supernatant-treated cells were low and not significantly different from untreated NBRC 15631 cultures (Fig. 2F and data not shown). These results suggest that the NBRC 15631 cells do not produce and/or sense autoinducer(s) but retain functional *luxO* and *luxR* genes that are epistatic to autoinducer sensing.

To assess the DS40M4 strain’s response to autoinducers, we performed supernatant-based assays with a strain of DS40M4 which contains deletions in all predicted autoinducer synthases. Strikingly, neither DS40M4 nor NBRC 15631 encodes a LuxM homolog, which synthesizes AI-1 in BB120. Thus, we constructed a Δ*luxS* Δ*cqsA* strain, which we predict lacks production of AI-2 and CAI-1, respectively. Cell-free supernatants from *V. campbellii* BB120 strains producing only AI-1, CAI-1, AI-2, combinations of two of the AIs, or all three autoinducers were incubated with the DS40M4 strain containing the P*_luxCDABE_-gfp* reporter plasmid, and GFP fluorescence was measured. We observed that DS40M4 responds to the presence of AI-2 and CAI-1 from BB120, but not AI-1 (Fig. 2E). Conversely, our positive-control series using a Δ*luxM* Δ*luxS* Δ*cqsA* BB120 strain responded synergistically to all three autoinducers, as expected (Fig. 2D). Both BB120 and DS40M4 have higher GFP expression in wild-type cultures than in cultures in which the synthases are deleted and supernatant is added, which suggests that the amount of autoinducers in supernatant-supplied cultures is smaller than in wild-type cultures (Fig. 2D and E). From these data, we conclude that DS40M4 has a quorum-sensing signaling system and LuxR and LuxO proteins that function similarly to BB120. However, DS40M4 does not sense AI-1 from BB120, and the lack of a LuxM homolog suggests that this branch of the signaling pathway is not conserved.

### Quorum sensing is not required for transformation in *V. campbellii* DS40M4 but is required in NBRC 15631.

**FIG 3:**
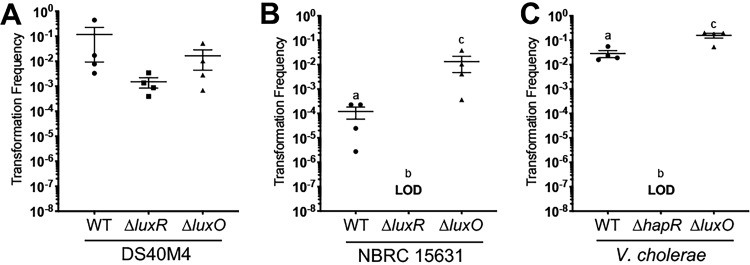
Natural transformation of *V. campbellii* DS40M4 does not require LuxR. (A and B) Transformation frequencies using chitin-independent transformation of a Δ*luxB*::Tm^r^ substrate (300 ng) into wild-type, Δ*luxR*, and Δ*luxO* strains of DS40M4 (A) and NBCR 15631 (B). LOD, limit of detection. (C) Transformation frequencies using chitin-independent transformation of a Δ*vc1807*::Tm^r^ substrate (300 ng) into wild-type, Δ*hapR*, and Δ*luxO* strains of V. cholerae. LOD, limit of detection. In panel A, there are no significant differences (ANOVA on log-transformed data; *P = *0.1054). In panels B and C, different letters indicate significant differences (ANOVA on log-transformed data, followed by Tukey’s multiple-comparison test; *P < *0.01).

In V. cholerae, HapR expression is required for natural transformation (8, 29). In the absence of *hapR*, deletion of *dns* results in modest increases in transformation (30). A *hapR* mutation can be bypassed by overexpression of *qstR* to produce transformants, though at a reduced rate compared to wild type (8). Combination of *qstR* expression and deletion of *dns* in a *hapR* mutant results in maximal transformation frequencies (10). To determine whether the *luxR* genes of DS40M4 and NBRC 15631 are required for natural transformation, we assayed transformation frequencies in Δ*luxR* and Δ*luxO* mutants compared to wild type for each *V. campbellii* strain using chitin-independent transformation. As a control, we performed the analogous experiment in V. cholerae with Δ*hapR* and Δ*luxO* mutants (Fig. 3C). Surprisingly, we observed that a Δ*luxR* mutant in the DS40M4 strain is capable of natural transformation at a frequency that is similar to wild type (Fig. 3A). The DS40M4 Δ*luxO* mutant also exhibits a similar transformation frequency as wild-type DS40M4 (Fig. 3A), which has been observed in V. cholerae (1). From these data, we conclude that quorum-sensing control of competence via LuxR regulation is not required for transformation in *V. campbellii* DS40M4.

Conversely, transformation of the Δ*luxR* NBRC 15631 strain does not yield antibiotic-resistant colonies. This result suggests that LuxR is required for transformation in NBRC 15631 (Fig. 3B). In addition, the wild-type level of transformation in NBRC 15631 is lower than in DS40M4 and V. cholerae. The Δ*luxO* NBRC 15631 strain has >100-fold-higher levels of transformation than the wild-type NBRC 15631 strain and frequencies similar to those observed in the DS40M4 Δ*luxO* strain (Fig. 3B). It is likely that the presumed high level of LuxR present in the Δ*luxO* strain restores transformation frequencies in NBRC 15631. Thus, we conclude that quorum sensing positively controls transformation in NBRC 15631.

### Conservation of competence genes and gene expression between BB120 and DS40M4.

**FIG 4:**
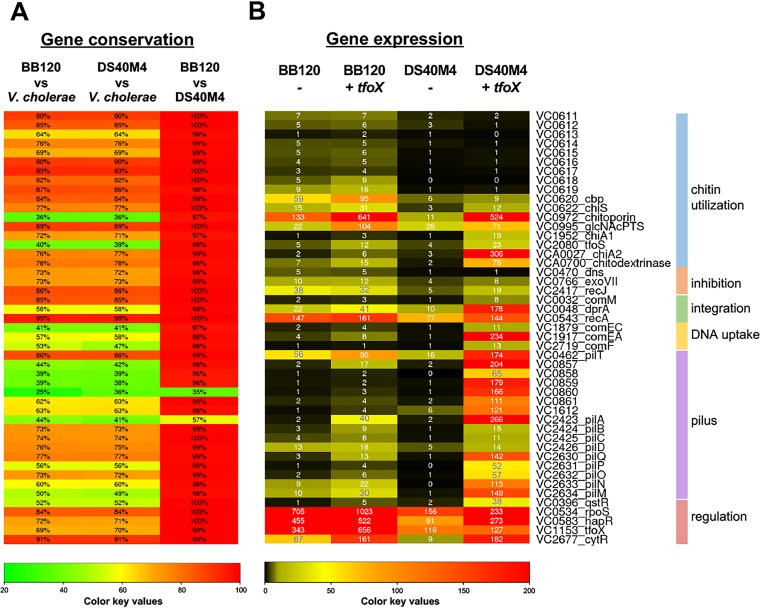
Comparative genomics and transcriptomics analyses of BB120 and DS40M4 competence genes. Genes required for DNA uptake and integration previously determined in V. cholerae were identified in BB120 and DS40M4 using reciprocal BLAST analyses. Genes are organized based on function. The locus tags correspond to V. cholerae genes; corresponding locus tags for BB120 and DS40M4 are in Table S4. (A) The chart indicates the amino acid identity shared between V. cholerae, BB120, and DS40M4, which is shown in each bar and by the color scale. (B) The chart indicates the RPKM values derived from RNA-seq data comparing either BB120 or DS40M4 transcript levels in the presence or absence of *tfoX* induction (strains contain plasmid pMMB67EH-tfoX-kanR).

To investigate the differences in transformation frequencies between the BB120 and DS40M4 *V. campbellii* strains, we performed a comparative genomics analysis of the known V. cholerae competence genes against the genes present in *V. campbellii* DS40M4 and BB120. We generated a list of 47 V. cholerae genes based on published data supporting a role for the gene product and/or transposon insertion sequencing (Tn-Seq) data suggesting that mutants lacking these genes exhibit differing phenotypes in natural transformation assays. Our analyses show that BB120 and DS40M4 both carry homologs of all known genes that play a role in competence in V. cholerae (Fig. 4A; Table S4). Conservation of amino acid identity ranges from 25% to 95% with a median value of 73%. However, the vast majority of genes that have low amino acid conservation with V. cholerae are still highly conserved between BB120 and DS40M4. For only two genes, *pilA* and *VC0860* (a minor pilin gene), there is low conservation among the three strains. For example, the *pilA* gene in BB120 shares 44% amino acid identity with V. cholerae *pilA* and only 57% identity with DS40M4 *pilA* (Fig. 4A).

10.1128/mBio.02788-19.8 TABLE S4 BB120 and DS40M4 homologs of V. cholerae competence genes. Download Table S4, DOCX file, 0.02 MB. Copyright © 2019 Simpson et al. 2019 Simpson et al. This content is distributed under the terms of the Creative Commons Attribution 4.0 International license.

Because it appears that BB120 carries all known genes required for competence at some degree of conservation, we next questioned whether the expression of these genes was sufficient for transformation. We performed transcriptome sequencing (RNA-seq) comparing gene expression in the presence or absence of *tfoX* induction in both BB120 and DS40M4 strains and analyzed the reads per kilobase of transcript per million mapped reads (RPKM). As expected, induction of TfoX expression resulted in upregulation of the genes required for DNA uptake and integration (*comEC*, *comEA*, *comM*, *comF*, and *dprA*) and pilus structure (*pilABCD*, *pilMNOPQ*, and minor pilins VC0857 to -0861) in DS40M4 (Fig. 4B). However, for most of these genes in BB120, there is no significant change in expression with or without *tfoX* induction (Fig. 4B). Further, even if an increase in gene expression was observed in BB120 with *tfoX* induction, it was modest compared to the large changes in gene expression in DS40M4 with *tfoX* induction. Collectively, these data suggest that expression levels of competence genes in BB120 are not sufficient for transformation. It is also possible that the *pilA* and *VC0860* homologs in BB120 are not functional, due to their lower conservation with DS40M4 homologs.

### Expression of QstR is necessary for natural transformation in DS40M4.

**FIG 5:**
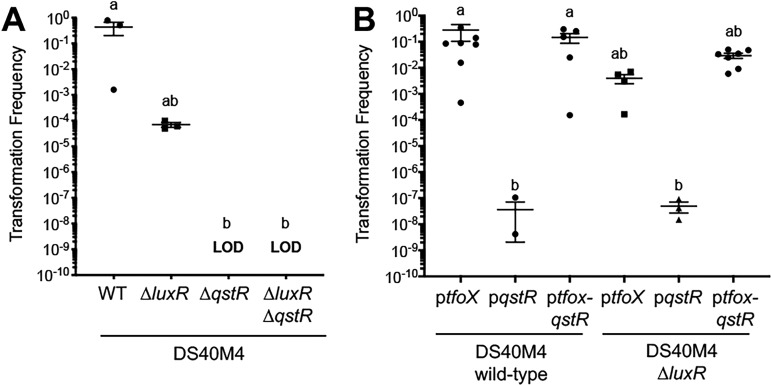
Endogenous QstR expression and ectopic TfoX overexpression are necessary for natural transformation. (A) Transformation frequencies using chitin-independent transformation of a Δ*luxB*::Spec^r^ substrate (300 ng) into wild-type, Δ*luxR*, Δ*qstR*, and Δ*luxR* Δ*qstR* strains of DS40M4, all containing pMMB67EH-tfoX-kanR. LOD, limit of detection. Different letters indicate significant differences (Kruskal-Wallis test; *P < *0.01). (B) Transformation frequencies using chitin-independent transformation of a Δ*luxB*::Tm^r^ substrate (300 ng) into wild-type and Δ*luxR* strains of DS40M4, either with the pMMB67EH-tfoX-kanR plasmid (p*tfoX*), the pCS39 plasmid (p*qstR*), or the pCS32 plasmid (p*tfoX-qstR*). Different letters indicate significant differences (Kruskal-Wallis test; *P < *0.01).

We next sought to examine whether DS40M4 requires QstR expression for competence, which is the case in V. cholerae. We observed that deletion of *qstR* in DS40M4 abolishes transformation, much like in V. cholerae, and this is epistatic to LuxR such that the Δ*luxR* Δ*qstR* deletion strain is also nontransformable (Fig. 5A). We also tested whether increased levels of *qstR* would increase transformation frequencies. We cloned the *qstR* gene from DS40M4 under the control of an IPTG-inducible promoter either alone or downstream of *tfoX* to form a synthetic operon, P*_tac_-tfoX-qstR*. We observed no significant changes in transformation frequencies in either wild-type or Δ*luxR* DS40M4 when *qstR* and *tfoX* are both expressed compared to *tfoX* expressed alone (Fig. 5B). Expression of *qstR* alone produced very low numbers of colonies (but they were measurable above the limit of detection), indicating that inducible expression of *tfoX* is necessary for high levels of transformation. The low transformation frequency in the absence of the inducible *tfoX* gene is likely a result of endogenous low-level expression of DS40M4 *tfoX*. From these data, we conclude that endogenous *qstR* and ectopic *tfoX* expression are both necessary to initiate high levels of natural transformation in *V. campbellii* DS40M4.

We also noted that the levels of *qstR* in BB120 are comparatively lower than in DS40M4 under *tfoX* induction conditions (normalizing compared to *recA*, Fig. 4B). Because *qstR* is required for expression of essential competence genes (e.g., *comEA* and *comEC*) in V. cholerae (10), we reasoned that the low levels of *qstR* expression in BB120 might be one reason for its inability to take up DNA. However, we did not observe any transformation in BB120 or HY01 under induction of both *tfoX* and *qstR* (data not shown).

### Natural transformation frequencies vary widely across *Vibrio* species.

**FIG 6:**
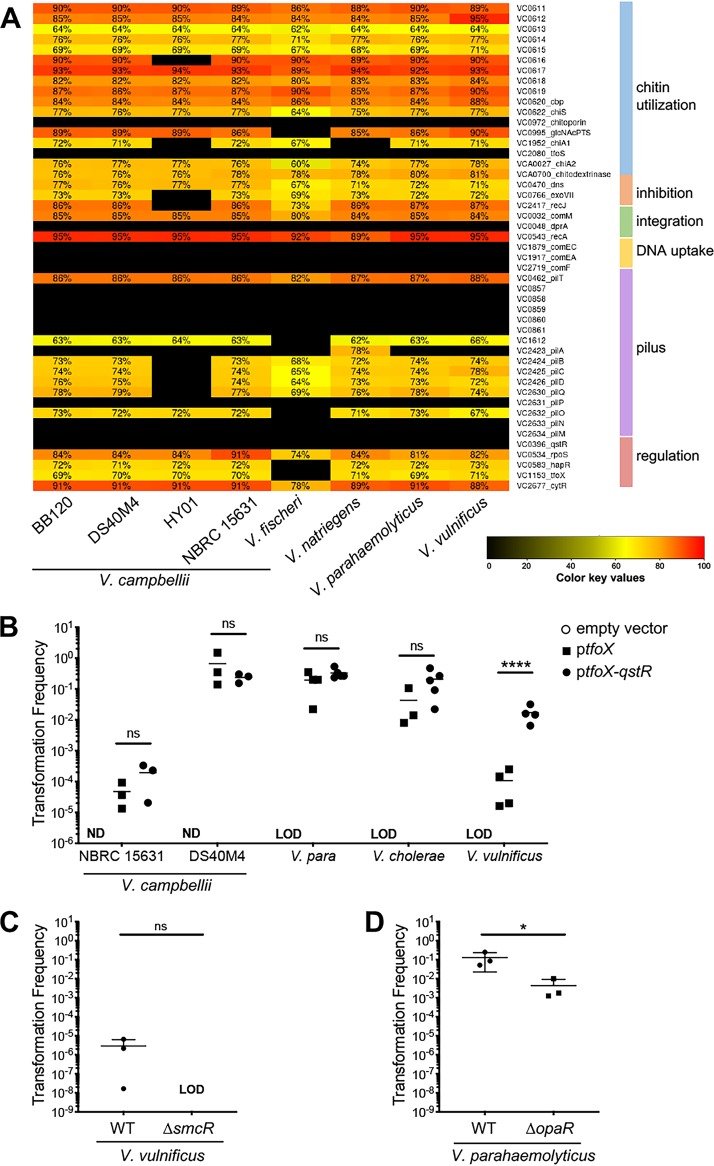
Natural transformation frequencies vary in *Vibrio* species. (A) Genes required for DNA uptake and integration previously determined in V. cholerae were identified in each *Vibrio* strain. Genes are organized based on function. The chart indicates the amino acid identity shared with V. cholerae proteins and indicated by the color scale. (B) Chitin-independent transformations in each of the listed *Vibrio* species using Spec^r^ linear tDNAs (300 ng) targeting the *luxR* homolog in each strain in the presence of a plasmid expressing either *tfoX* alone (pMMB67EH-tfoX-kanR) or both *tfoX* and *qstR* (pCS32). Asterisks indicate *P < *0.0001 (ANOVA on log-transformed data followed by Tukey’s multiple-comparison test) for all strains except V. parahaemolyticus (Kruskal-Wallis test, *P < *0.01). ns, not significant. LOD, limit of detection. ND, not determined. (C and D) Transformation frequencies using chitin-independent transformation of a Δ*pomB*::Tm^r^ substrate (300 ng) into wild-type and Δ*smcR* V. vulnificus strains expressing *tfoX* (pMMB67EH-tfoX-kanR) (C) or wild-type and Δ*opaR* V. parahaemolyticus strains expressing *tfoX* (pMMB67EH-tfoX-kanR) (D). LOD, limit of detection. For both panels A and B, asterisks indicate significant differences (unpaired *t* test; *P < *0.05).

To investigate the differences in transformation capabilities between various *Vibrio* strains and species, we performed a comparative genomics analysis of the known V. cholerae competence genes against the genes present in *V. campbellii*, V. parahaemolyticus, Vibrio fischeri, and V. vulnificus. For these clusters, we chose a minimum percent identity requirement of 60% to decrease noise and increase confidence in functional similarity, and thus any proteins with less than 60% amino acid identity appear blank (Fig. 6A). The results indicate that (i) many competence genes are highly conserved across species, including more distantly related species such as V. fischeri, and (ii) absence of competence gene conservation does not correlate with lack of transformation. For example, the minor pilins and ComEC/ComEA are not well conserved in any vibrio that we analyzed, yet transformation has been shown for several of these species. Thus, we conclude that a strain’s transformation capability cannot be predicted from gene conservation.

The notable differences in transformation capabilities despite high conservation of competence genes between strains of *V. campbellii* led us to investigate the frequencies of natural transformation in other vibrios. Varied transformation frequencies have been reported for other *Vibrio* species in the literature, with *V. natriegens* having the highest level of transformation frequency (10, 12, 14, 16, 18, 19, 31). However, several of these experiments were performed in different labs with different conditions, such as different *tfoX* genes, tDNA homology lengths, tDNA quantity, media, and outgrowth times. To formally assay frequencies of transformation under consistent experimental conditions, we tested transformation using both chitin-dependent and -independent methods in multiple *Vibrio* species: *V. campbellii*, V. parahaemolyticus, V. cholerae, and V. vulnificus. The tDNA substrates were generated to target the gene encoding the LuxR/HapR homolog in each strain: *luxR*, *opaR*, *hapR*, and *smcR*, respectively. In our experiment using chitin from shrimp shells, only V. cholerae was able to undergo natural transformation when chitin was used to induce competence (Fig. S3B).

When we stimulated competence by overexpressing TfoX (chitin independent), all strains except *V. campbellii* BB120 and HY01 were able to take up the tDNA and produce recombinants (Fig. 6B). V. vulnificus exhibited the lowest transformation frequency, and *V. campbellii* DS40M4 had the highest frequencies (Fig. 1B and Fig. 6B). Similarly to a previously published comparison, V. parahaemolyticus has a similar transformation frequency as V. cholerae (32). Because expression of *qstR* is necessary for transformation in V. cholerae and *V. campbellii* DS40M4 and levels of *qstR* varied between BB120 and DS40M4, we hypothesized that the low transformation rate of V. vulnificus might be due to inadequate *qstR* expression. We introduced the same P*_tac_-tfoX-qstR* plasmid pCS32 into each *Vibrio* strain tested above and assayed for transformation frequency. We observed a >2-log increase in transformation in V. vulnificus (Fig. 6B), though there were no significant increases in the other vibrios tested. This is a noteworthy improvement of transformation frequency in V. vulnificus, which is otherwise too low to use the genetic technique called multiplex genome editing by natural transformation (MuGENT) that relies on high transformation frequencies to introduce multiple mutations simultaneously (31).

### Quorum sensing is not required for transformation in V. parahaemolyticus.

To determine if the lack of requirement for LuxR for competence extends beyond *V. campbellii*, we tested transformation in V. vulnificus and V. parahaemolyticus in the presence or absence of the LuxR-type quorum-sensing regulator in each species. Deletion of the *smcR* gene in V. vulnificus abolished transformation, similar to what is observed in V. cholerae (Fig. 6C). However, deletion of the *opaR* gene in V. parahaemolyticus did not eliminate transformation but significantly decreased it ∼30-fold, similar to what is observed in *V. campbellii* DS40M4 (Fig. 3A and Fig. 6D). These results show that quorum sensing and competence are linked but not required in V. parahaemolyticus.

## DISCUSSION

We identified two strains of *V. campbellii* that are capable of natural transformation in the presence of *tfoX* expression. We observed a wide diversity in transformation frequencies, not only between *Vibrio* species but also among *V. campbellii* strains. The strain-strain variation supersedes any species-level variation such that some *V. campbellii* strains are highly competent and some are incapable of transformation under tested laboratory conditions. For example, the lack of transformation observed in the highly pathogenic HY01 strain compared to the high frequencies observed in the oceanic isolate DS40M4 underscores a possible selective pressure driving conservation of competence functions. As proposed in a recent study (10), it is possible that vibrios that are adapted to specific niches, such as those of a host organism for pathogenic strains, might have lost the ability to take up DNA if it were no longer beneficial. Conversely, natural transformation might be more advantageous for strains existing free-living in the ocean that are more likely to be in close proximity to other strains and benefit from the uptake of new DNA sequences. Though it is interesting to speculate why the strain-strain variation is so strong, our small data set of four *V. campbellii* strains is not sufficient to draw conclusions.

It also appears that differences in transformation frequencies may be reflective of strains’ evolutionary relatedness. V. parahaemolyticus and *V. campbellii* are more closely related to each other than to V. cholerae or V. vulnificus (Fig. 1A), and our data suggest that at least one *V. campbellii* strain and one V. parahaemolyticus strain are naturally transformable in the absence of quorum sensing. Initially, we had hoped to glean more information about *Vibrio* transformation rates by examining available metagenomics data for key genes. However, our comparative genomics analyses have shown that a strain’s frequency of natural transformation (or lack thereof) cannot be determined by the presence or absence (or conservation level) of a gene or set of genes. Rather, even the most closely related strains in our small data set had large differences in transformation rates (i.e., BB120 and DS40M4). Instead, the levels of expression of the competence genes appear to be a better predictor of transformation capability.

We determined that the genes encoding the quorum-sensing system proteins LuxR and LuxO are conserved in DS40M4 and NBRC 15631 and function similarly to BB120. However, it is intriguing that the NBRC 15631 strain produced low levels of GFP expression in our quorum-sensing reporter assay. Because we used a *luxCDABE-gfp* reporter assay, we expected that GFP would be activated when cells reached high cell density, which is what was observed in BB120 and DS40M4. Conversely, the wild-type NBRC 15631 cells do not express GFP at high densities, but only when *luxO* is deleted, and this is dependent on LuxR. We suspect that there is a defect in regulation in the quorum-sensing pathway of NBRC 15631 that results in a low-cell-density-like state but that can be bypassed by deletion of *luxO*. In this low-cell-density state, low levels of LuxR would be produced, which is likely the cause of the lower transformation frequencies in wild-type NBRC 15631 compared to DS40M4. We also showed that the quorum-sensing circuit in DS40M4 functions similarly to BB120, with the exception of the AI-1-sensing system. Because DS40M4 lacks a LuxM homolog and the strain contains a LuxN protein that does not respond to AI-1 from BB120, we hypothesize that the AI-1 system is nonfunctional. However, the AI-2 and CAI-1 systems responded to exogenous autoinducers produced by BB120 in a manner similar to BB120, suggesting that DS40M4 recognizes the same molecules.

Our genomic and transcriptomic comparisons of DS40M4 and BB120 were both vital in understanding the difference(s) between these strains and V. cholerae. Overexpression of V. cholerae *tfoX* in *V. campbellii* DS40M4 produced an expression profile for the 47 competence genes similar to the profile produced in V. cholerae upon *tfoX* induction (33). One exception to this is that we observed an ∼10-fold increase in *pilT* expression with *tfoX* induction, compared to V. cholerae, which showed similar levels of *pilT* expression in the presence and absence of *tfoX* induction (33). Another exception is that *tfoX* induction in DS40M4 resulted in an ∼20-fold increase in *cytR* expression in DS40M4, whereas in a similar experiment in V. cholerae, *cytR* expression was increased only ∼4-fold (33). Although the reason for these differences is unclear, it minimally indicates that there are slight differences in the TfoX regulons of *V. campbellii* DSS40M4 and V. cholerae. Overall, our observation that DS40M4 upregulates >20 genes similarly to V. cholerae in the presence of TfoX suggests high conservation of the regulatory network controlling competence.

**FIG 7:**
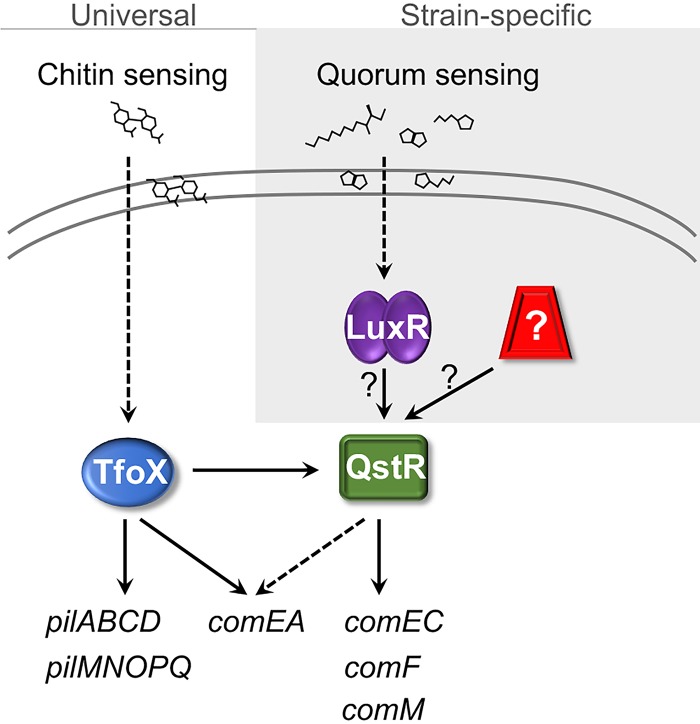
Model for regulation of natural transformation in *Vibrio* species. In some *Vibrio* strains, both chitin-sensing and quorum-sensing systems are required to activate TfoX and LuxR-type proteins, respectively. Conversely, some *Vibrio* strains do not require LuxR proteins, and the influence of quorum sensing on natural transformation is minimal at most. We propose that all *Vibrio* strains utilize TfoX and QstR to drive expression of genes required for competence, but the signaling systems used to regulate QstR differ.

Our data suggest that the two known signaling systems—chitin sensing and quorum sensing—have differing effects on transformation frequencies among *Vibrio* strains (Fig. 7). The high success rate of TfoX-dependent transformation in multiple *Vibrio* species suggests that this aspect of the regulatory network is conserved in all *Vibrio* species. Chitin-based induction of TfoX expression has been shown for multiple *Vibrio* species, suggesting that this is a conserved mechanism to stimulate TfoX induction. Lack of transformation by the chitin-dependent method in the lab does not mean that chitin cannot induce TfoX but may mean that other factors or components may be required. Thus, we speculate that the chitin-sensing system is universal in vibrios. Conversely, we have shown that quorum-sensing induction of QstR is not required in some strains. Deletion of *luxR/opaR* in *V. campbellii* DS40M4 and V. parahaemolyticus minimally affected transformation, and these mutant strains retain relatively high transformation frequencies that are orders of magnitude better than transformation in V. vulnificus. It is possible that either QstR is constitutively active in these strains or another signaling system drives expression of QstR (Fig. 7). Examination of *qstR* expression under various conditions in these strains will aid in determining whether other regulatory proteins play a role in natural transformation.

Competence in bacteria has been observed in more than 40 diverse species of bacteria (34). Our finding that conservation of the regulatory proteins governing competence varies—but not the functional competence genes themselves—is reminiscent of what is observed in Gram-positive bacteria. For example, in *Streptococcus* and *Bacillus*, the timing of competence gene expression and the proteins in the regulatory network are species specific (35). Specifically, in *Streptococcus* species, quorum sensing (competence signaling peptides [CSPs]) plays important roles in expression of competence genes (36). Among different pherotypic groups of streptococci, there is strain specificity in the sequence of the CSPs, the regulatory genes, and the functionality of the competence system (37). For those strains of streptococci that have not been shown to be competent, it is likely that some of these are transformable, but the conditions that drive competence are unknown (38). Thus, our observations in vibrios are remarkably similar in that the regulatory networks vary at the strain level, but competence genes are present throughout the genus (Fig. 7).

Unfortunately, we were unsuccessful at restoring competence to *V. campbellii* BB120. It is likely that the limitations to transformation in BB120 are multifactorial, including a potentially defective PilA and/or VC0860 minor pilin homolog; decreased gene expression of genes required for pilus assembly, DNA uptake, and DNA integration upon induction of *tfoX*; and low expression of QstR. PilA sequences vary widely among environmental V. cholerae strains (39), and it was recently shown that differences in PilA protein sequences among strains enable V. cholerae cells to discriminate between each other, which leads to decreased cell aggregation (40). Thus, it is unsurprising that the PilA sequence differs between all three vibrio strains (Fig. 4A). However, the VC0860 minor pilin protein is required for competence in V. cholerae (41), and thus, the low conservation of this gene between BB120 and DS40M4 may reduce or eliminate transformation. It is also not clear why so many competence genes failed to induce upon TfoX expression, whereas this method of inducing competence was highly successful in *V. campbellii* DS40M4 and NBRC 15631 and other *Vibrio* species. It is possible that the variability in response to the V. cholerae TfoX may drive some of the differences in transformation frequencies observed in the other *Vibrio* strains. We postulate that either V. cholerae TfoX does not function properly in BB120 or BB120 lacks regulatory control of the core competence genes from another transcription factor, possibly one that is not known in V. cholerae yet. The only BB120 gene known to be controlled by TfoX that responded with its induction was *qstR*, suggesting that TfoX might function in BB120 but perhaps at a reduced capability. This is in accordance with the finding that V. fischeri *tfoX* is slightly decreased in the ability to cross-activate the *qstR* of V. cholerae (32). One additional possibility is that there is a regulatory feedback loop that downregulates transcription levels of structural genes in the absence of a functional pilus (i.e., if the BB120 VC0860 minor pilin protein is not functional), similar to the homeostatic regulation of flagellin in *Bacillus* (42). Future experiments are required to dissect the broken pieces of the BB120 competence regulatory and functional networks to synthetically generate competence in this model organism.

## MATERIALS AND METHODS

### Bacterial strains and media.

All bacterial strains and plasmids used are listed in Table S1 in the supplemental material. Escherichia coli was grown in lysogeny broth (LB) for all experiments at 37°C. V. cholerae was grown in LB at 30°C. V. parahaemolyticus was grown at 30°C either on LBv2 (LB medium supplemented with additional 200 mM NaCl, 23.14 mM MgCl_2_, and 4.2 mM KCl) for chitin-independent transformations or on LMv3 (LB medium supplemented with additional 513 mM NaCl) for chitin-dependent transformations. *V. campbellii*, *V. natriegens*, and V. vulnificus were grown at 30°C on Luria marine (LM) medium for chitin-dependent transformations (LB medium supplemented with additional 171 mM NaCl) or LBv2 for chitin-independent transformations. Instant ocean water (IOW) medium was used in the chitin-dependent and -independent transformations; it consists of Instant Ocean sea salts (Aquarium Systems, Inc.) diluted in sterile water (0.5× = 7 g/liter, 2× = 28 g/liter). IOW at 0.5× was used for V. cholerae, and IOW at 2× was used for all other strains. Chitin slurry consists of 8 g of chitin powder from shrimp shells (Sigma-Aldrich) in 150 ml of 0.5× IOW. When necessary, medium was supplemented with carbenicillin (100 μg/ml), kanamycin (Kan; 50 μg/ml for E. coli, 100 μg/ml for vibrios), spectinomycin (200 μg/ml), chloramphenicol (10 μg/ml), and/or trimethoprim (10 μg/ml). Plasmids were transferred from E. coli to *Vibrio* strains by conjugation on LB plates. Exconjugants were selected on LB or LM plates with polymyxin B at 50 U/ml and the appropriate selective antibiotic.

10.1128/mBio.02788-19.5 TABLE S1 Strains used in this study. Download Table S1, DOCX file, 0.03 MB. Copyright © 2019 Simpson et al. 2019 Simpson et al. This content is distributed under the terms of the Creative Commons Attribution 4.0 International license.

### Molecular methods.

All PCR products were synthesized using Phusion HF polymerase (New England Biolabs). Sequencing of constructs and strains was performed at Eurofins Scientific. Cloning procedures and related protocols are available upon request. Oligonucleotides used in the study are listed in Table S3. Linear tDNAs were generated by splicing-by-overlap-extension (SOE) PCR as previously described by Dalia et al. (17).

10.1128/mBio.02788-19.7 TABLE S3 Oligonucleotides used in this study. Download Table S3, DOCX file, 0.02 MB. Copyright © 2019 Simpson et al. 2019 Simpson et al. This content is distributed under the terms of the Creative Commons Attribution 4.0 International license.

### Natural transformation.

Chitin-independent transformations were performed according to the protocol established in the work of Dalia et al. (19). Transformation frequency was calculated as the number of antibiotic-resistant colonies divided by viable cells, and these results are graphed on the *y* axis in all graphs. Chitin-dependent transformations were performed according to the protocol established in the work of Dalia (22). Following natural transformation, strains containing the correct target mutation were identified via colony PCR with a forward and reverse detection primer (Table S3).

### Statistical analyses.

All data were analyzed using log-transformed data. We tested for skewness and kurtosis before proceeding with analysis of variance (ANOVA). In experiments with results of zero (no transformation, no colonies), a constant (1.0 × 10^−8^) was added to all results and those data were log transformed. The residuals were examined for each ANOVA to ensure that they had a constant variance. For data sets not matching these criteria, we performed nonparametric tests to compare means between groups.

### GFP expression assays.

*Vibrio* strains were first cured of the pMMB67EH-tfoX-kanR plasmid by serial inoculation (3 to 4 times) in the absence of antibiotic selection. The pCS19 reporter plasmid was conjugated into strains. Overnight cultures were diluted 1:5,000 into fresh medium and incubated at 30°C with shaking overnight. GFP fluorescence and OD_600_ were measured in black 96-well plates using the BioTek Cytation 3 plate reader. For assays in which supernatants were added, overnight cultures were centrifuged to pellet cells for 1 min at 13,000 rpm, supernatants were filtered through an 0.22-μm filter, and these were added to fresh LM plus Kan medium at 20% final concentration. Cells from strains to be assayed in the presence of supernatants were added to these supplemented media at a 1:5,000 dilution of an overnight culture and incubated overnight with shaking at 30°C. GFP and OD_600_ were measured as described above.

### RNA-seq.

Strains were inoculated in 5 ml LBv2 and grown overnight shaking at 250 rpm at 30°C. Each strain was back-diluted to an OD_600_ of 0.005 in fresh LBv2 (uninduced control samples) or LBv2 with 100 μM IPTG (induced samples). Cultures were grown shaking at 250 rpm at 30°C until they reached an OD_600_ of ∼1.8. Cells were collected by centrifugation at 3,700 rpm at 4°C for 10 min. The supernatant was removed, and the pellets were flash frozen in liquid N_2_ and stored at −80°C. RNA was isolated using the Qiagen RNeasy Mini kit according to the manufacturer’s protocol and treated with DNase (Ambion). RNA-seq was performed at the Center for Genomics and Bioinformatics at Indiana University. Isolated RNA was treated with the Ribo-Zero rRNA (bacteria) removal kit (Illumina). Purified RNA was prepared using the TruSeq Stranded mRNA HT sample prep kit (Illumina) according to the manufacturer’s protocols; dual-indexed adapters were added to libraries for multiplexing, and then libraries were cleaned by AMPure XP beads (Beckman Coulter).

Sequencing reads were trimmed using Trimmomatic (version 0.38 [43]) with a minimum trimmed read length of 30. The trimmed reads were mapped on to the Vibrio campbellii BB120 genome using Bowtie 2 (version 2.3.4.3 with default parameters [44]). Read counts for genes and intergenic intervals were calculated using a custom perl script. Resulting gene/interval counts were used to conduct differential expression analysis using the program DESeq2 algorithm with default parameters (45).

### Reciprocal BLAST.

Genome sequences and annotated protein sequences for *V. campbellii* strains BB120 (accession numbers CP000789.1, CP000790.1, and CP000791.1) and DS40M4 (accession numbers CP030788.1, CP030789.1, and CP030790.1) were obtained from GenBank. To ensure that we did not miss any unannotated genes, the genome sequences were reannotated with Prokka ver. 1.12 (46) (parameters: -minpid 70 -usegenus -hmmlist TIGRFAM, CLUSTERS, Pfam, HAMAP). Protein sequences encoded by annotated genes from the following strains were used as the training set for Prokka predictions: *V. campbellii* strains BB120 (ATCC BAA-1116), DS40M4, HY01, and NBRC 15631 (ATCC 25920, CAIM 519); V. cholerae N16961; V. fischeri ES114; *V. natriegens* NBRC 15636 (ATCC 14048); V. parahaemolyticus RIMD 2210633; and V. vulnificus ATCC 27562. Protein sequence sets for each of the two *V. campbellii* strains BB120 and DS40M4 were prepared by combining sequences from GenBank annotation with those from Prokka predictions. The two protein sequence sets were aligned against each other using NCBI BLASTP ver. 2.7.1. For all the sequences in each set, the best-scoring matches in the other set were identified based on the highest bit score. When multiple best hits were encountered, the GenBank annotated gene maintaining synteny with the best hits for the neighboring genes was preferred over the rest. Orthologous gene pairs for the two strains were called when the protein sequences encoded by the two genes identified each other as their best match.

### Analysis of *Vibrio* competence genes.

Protein sequence sets for the nine *Vibrio* strains, each comprising sequences from GenBank annotation and those from Prokka predictions (described above under “Reciprocal BLAST”), were prepared and combined into a single sequence set. This combined protein sequence set was clustered using cd-hit ver. 4.6 (47) with the minimum sequence identity cutoff set at 60% (parameters: -g 1 -s 0.8 -n 3). The clusters associated with competence genes from V. cholerae were obtained, and the protein sequences for the top hits from each species within each cluster were extracted and tested with multiple sequence alignment.

### Phylogenetic construction.

Nucleotide sequences from the coding regions for the annotated genes were downloaded from GenBank for the nine *Vibrio* species. The sequences from *V. campbellii* BB120 were aligned against those from the remaining eight genomes. The core genome was defined by identifying genes present in all nine *Vibrio* strains with at least 70% DNA identity over at least 80% of the total length of the corresponding homologous gene in BB120 using the method previously described (48). This formed the core set of 79 genes specific to these nine *Vibrio* strains. The protein sequences for the core gene set across all the nine genomes were obtained, and a multiple sequence alignment was performed separately for each gene using MUSCLE ver. 3.8.31 (49). After trimming the ends of the alignments to remove contiguous gaps (if any) resulting from unequal gene lengths, the alignments from each gene for each species were concatenated in order. Using this combined amino acid multiple sequence alignment file as input to the program RAxML ver. 8.2.12 (50), a best-scoring maximum likelihood tree was constructed.

### Data availability.

The RNA-seq results were deposited in the National Center for Biotechnology Information Gene Expression Omnibus database (NCBI GEO) with accession number GSE136941.

10.1128/mBio.02788-19.1 TEXT S1 Supplemental references. Download Text S1, DOCX file, 0.01 MB. Copyright © 2019 Simpson et al. 2019 Simpson et al. This content is distributed under the terms of the Creative Commons Attribution 4.0 International license.
