# Supplementary figures and images for: Diversity in Natural Transformation Frequencies and Regulation across Vibrio Species
Source: mBio. 2019 Dec 17;10(6):e02788-19. doi: 10.1128/mBio.02788-19 (PMC6918086; doi:10.1128/mBio.02788-19)

Figure S1

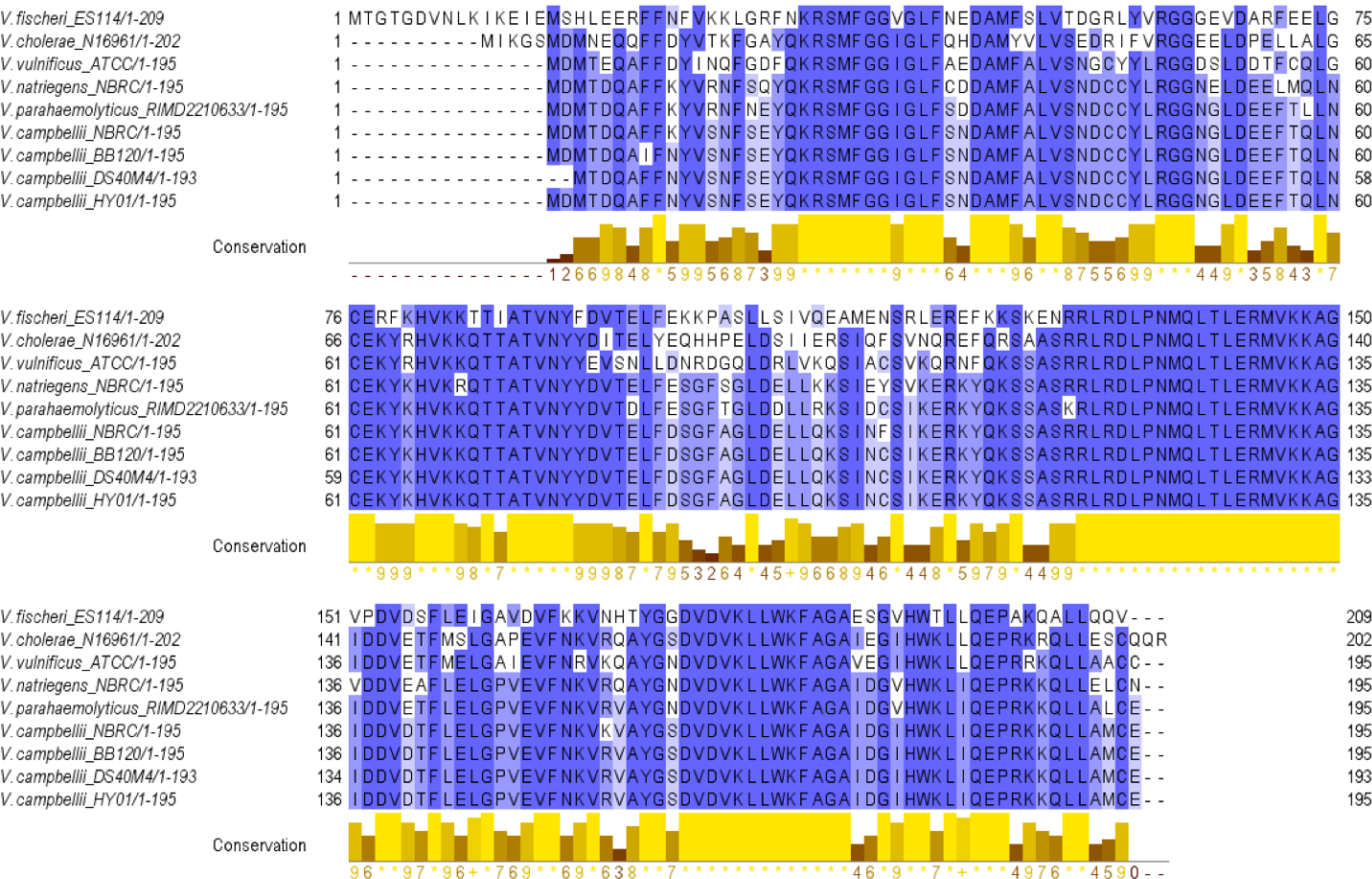

Supplement: FIG S1 [file mBio.02788-19-sf001.pdf]

**Figure S2**

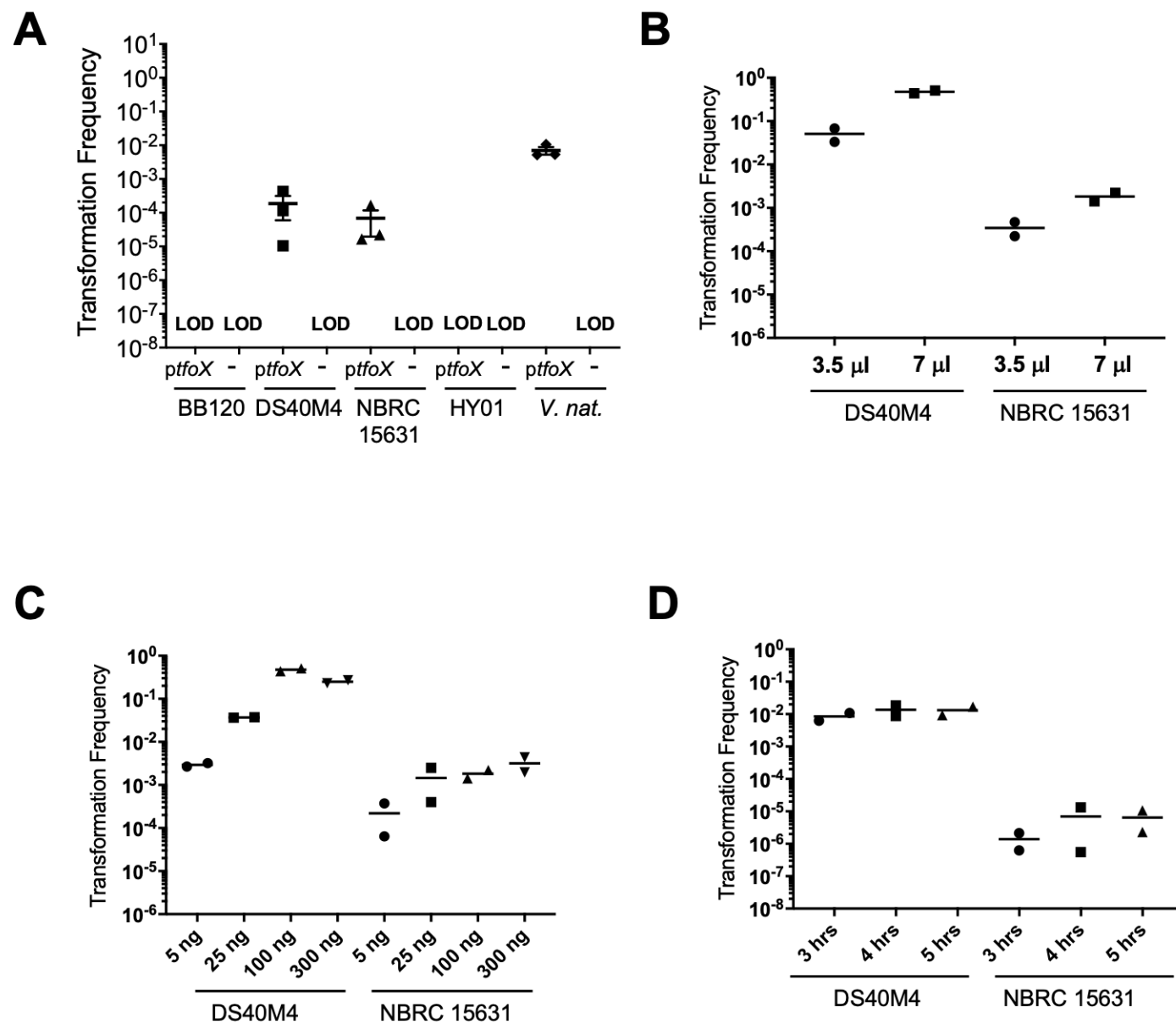

Supplement: FIG S2 [file mBio.02788-19-sf002.pdf]

**Figure S3**

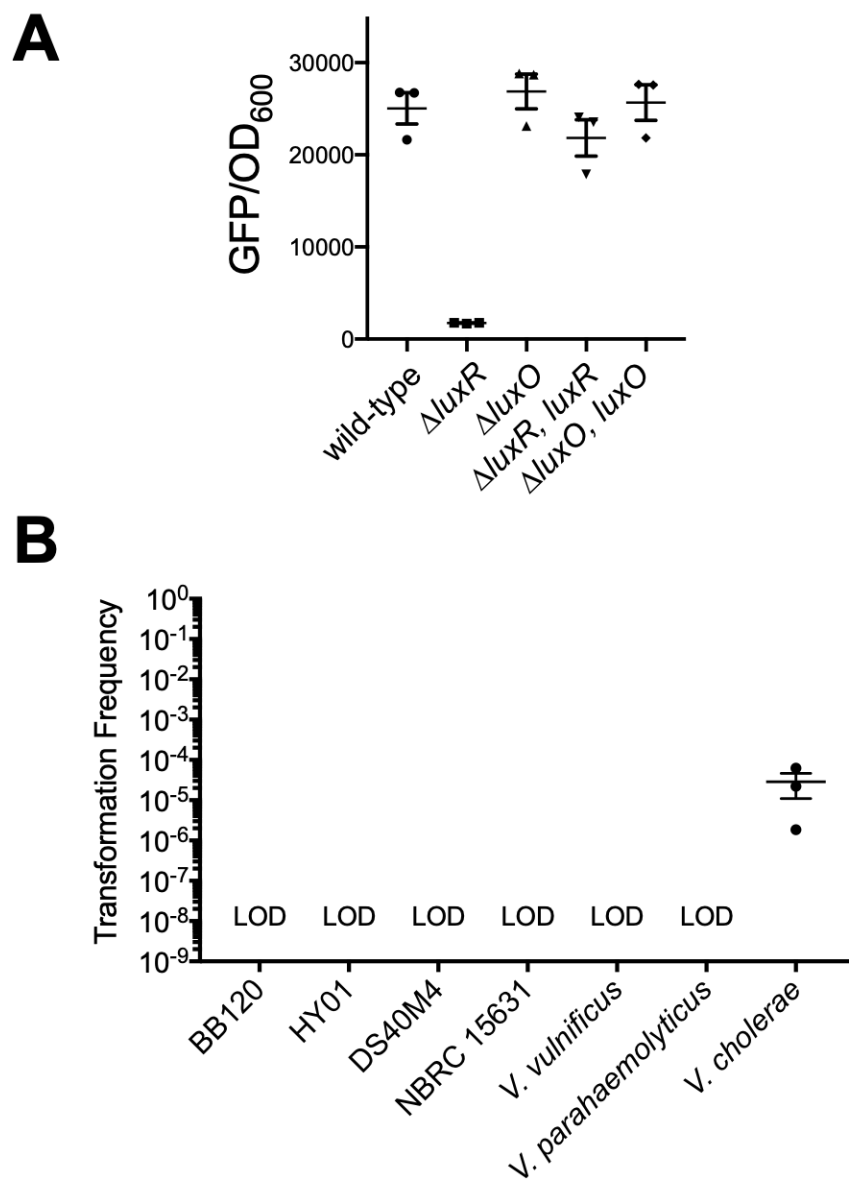

Supplement: FIG S3 [file mBio.02788-19-sf003.pdf]
