# Supplementary material for: Diversity in Natural Transformation Frequencies and Regulation across Vibrio Species
Source: mBio. 2019 Dec 17;10(6):e02788-19. doi: 10.1128/mBio.02788-19 (PMC6918086; doi:10.1128/mBio.02788-19)
Supplement: TABLE S2 [file mBio.02788-19-st002.docx]

**Table S2.** Plasmids used in this study.

| Name | Description | Reference |
| --- | --- | --- |
| pMMB67EH-tfoX  pMMB-pLuxC-GFP | P*_tac_::tfoX*; *amp^R^* | (10) |
| pMMB67EH | Empty vector control; *amp^R^* | (12) |
| pMMB67EH-tfoX-kanR k | Derivative of pMMB67EH-tfoX; *kan^R^* | This study |
| pMMB67EH-kanR | Empty vector control; *kan^R^* | This study |
| pCS19 | P*_luxCDABE_::gfp*; *kan^R^*; derivative of pMMB67EH-tfoX-kanR | This study |
| pJV298 | P*_tac_-gfp*; *lacIq*; colE1 origin, *CM^R^* | This study |
| pCS32 | P*_tac_::tfoX-qstR*; *kan^R^*; derivative of pMMB67EH-tfoX-kanR | This study |
| pCS39 | pMMB-QstR | This study |
