# Supplementary material for: Diversity in Natural Transformation Frequencies and Regulation across Vibrio Species
Source: mBio. 2019 Dec 17;10(6):e02788-19. doi: 10.1128/mBio.02788-19 (PMC6918086; doi:10.1128/mBio.02788-19)
Supplement: TABLE S4 [file mBio.02788-19-st004.docx]

**Table S4.** BB120 and DS40M4 homologs of *V. cholerae* competence genes.

| *V. cholerae* locus tag | DS40M4 locus tag | BB120 locus tag | Gene name | Competence category |
| --- | --- | --- | --- | --- |
| VC0611 | DSB67_12495 | VIBHAR_03432 |  | chitin |
| VC0612 | DSB67_12490 | VIBHAR_03431 |  | chitin |
| VC0613 | DSB67_12485 | VIBHAR_03430 |  | chitin |
| VC0614 | DSB67_12480 | VIBHAR_03429 |  | chitin |
| VC0615 | DSB67_12475 | VIBHAR_03428 |  | chitin |
| VC0616 | DSB67_12470 | VIBHAR_03427 |  | chitin |
| VC0617 | DSB67_12465 | VIBHAR_03426 |  | chitin |
| VC0618 | DSB67_12460 | VIBHAR_03425 |  | chitin |
| VC0619 | DSB67_12455 | VIBHAR_03424 |  | chitin |
| VC0620 | DSB67_12450 | VIBHAR_03423 | *cbp* | chitin |
| VC0622 | DSB67_12445 | VIBHAR_03422 | *chiS* | chitin |
| VC0972 | DSB67_03685 | VIBHAR_01269 | chitoporin | chitin |
| VC0995 | DSB67_03995 | VIBHAR_01336 | glcNAcPTS | chitin |
| VC1952 | DSB67_11840 | VIBHAR_03258 | *chiA1* | chitin |
| VC2080 | DSB67_04120 | VIBHAR_01362 | *tfoS* | chitin |
| VCA0027 | DSB67_19440 | VIBHAR_06955 | *chiA2* | chitin |
| VCA0700 | DSB67_23890 | VIBHAR_05945 | chitodextrinase | chitin |
| VC0470 | DSB67_13165 | VIBHAR_03571 | *dns* | inhibition |
| VC0766 | DSB67_02900 | VIBHAR_01075 | *exoVII* | inhibition |
| VC2417 | DSB67_02380 | VIBHAR_00958 | *recJ* | inhibition |
| VC0032 | DSB67_15695 | VIBHAR_00409 | *comM* | integration |
| VC0048 | DSB67_15605 | VIBHAR_00389 | *dprA* | integration |
| VC0543 | DSB67_12880 | VIBHAR_03513 | *recA* | integration |
| VC1879 | DSB67_04755 | VIBHAR_01534 | *comEC* | integration |
| VC1917 | DSB67_04445 | VIBHAR_01422 | *comEA* | integration |
| VC2719 | DSB67_00645 | VIBHAR_00615 | *comF* | integration |
| VC0462 | DSB67_13195 | VIBHAR_03577 | *pilT* | pilus |
| VC0857 | DSB67_03055 | VIBHAR_01136 |  | pilus |
| VC0858 | DSB67_03060 | VIBHAR_01137 |  | pilus |
| VC0859 | DSB67_03065 | VIBHAR_01138 |  | pilus |
| VC0860 | DSB67_03070 | VIBHAR_01139 |  | pilus |
| VC0861 | DSB67_03075 | VIBHAR_01140 |  | pilus |
| VC1612 | DSB67_08830 | VIBHAR_02493 |  | pilus |
| VC2423 | DSB67_12665 | VIBHAR_03468 | *pilA* | pilus |
| VC2424 | DSB67_12670 | VIBHAR_03469 | *pilB* | pilus |
| VC2425 | DSB67_12675 | VIBHAR_03470 | *pilC* | pilus |
| VC2426 | DSB67_12680 | VIBHAR_03471 | *pilD* | pilus |
| VC2630 | DSB67_13985 | VIBHAR_00029 | *pilQ* | pilus |
| VC2631 | DSB67_13990 | VIBHAR_00030 | *pilP* | pilus |
| VC2632 | DSB67_13995 | VIBHAR_00031 | *pilO* | pilus |
| VC2633 | DSB67_14000 | VIBHAR_00032 | *pilN* | pilus |
| VC2634 | DSB67_14005 | VIBHAR_00033 | *pilM* | pilus |
| VC0396 | DSB67_13770 | VIBHAR_03706 | *qstR* | regulation |
| VC0534 | DSB67_12895 | VIBHAR_03517 | *rpoS* | regulation |
| VC0583 | DSB67_12620 | VIBHAR_03459 | *hapR* | regulation |
| VC1153 | DSB67_06270 | VIBHAR_02628 | *tfoX* | regulation |
| VC2677 | DSB67_01305 | VIBHAR_00726 | *cytR* | regulation |
