# Supplementary material for: Diversity in Natural Transformation Frequencies and Regulation across Vibrio Species
Source: mBio. 2019 Dec 17;10(6):e02788-19. doi: 10.1128/mBio.02788-19 (PMC6918086; doi:10.1128/mBio.02788-19)
Supplement: TABLE S1 [file mBio.02788-19-st001.docx]

**Table S1.** Strains used in this study.

| Strains | Genotype | Reference |
| --- | --- | --- |
| ***V. campbellii* strains** |  |  |
| BB120 | wild-type (ATCC BAA-1116) | (1) |
| JAF78 | BB120 Δ*luxO::CM^R^* | (2) |
| CAS156 | BB120 Δ*luxO::CM^R^*, pCS19 | This study |
| KM669 | Δ*luxR* | (3) |
| CAS094 | BB120 Δ*luxR*, pCS19 | This study |
| CAS093 | BB120, pCS19 | This study |
| CAS190 | BB120, pCS32 | This study |
| CAS003 | BB120, pMMB67EH-tfox-kanR | This study |
| CAS165 | BB120, pMMB67EH-kanR | This study |
| DS4M04 | wild-type | (4) |
| CAS141 | DS40M4 Δ*luxO::Spec^R^* | This study |
| CAS187 | DS40M4 Δ*luxO::Spec^R^* Δ*luxB::luxO:: TM^R^* | This study |
| CAS189 | DS40M4 Δ*luxO::Spec^R^* Δ*luxB::luxO:: TM^R^*, pCS19 | This study |
| CAS179 | DS40M4 Δ*luxO::Spec^R^* Δ*luxB::luxO:: TM^R^*, pMMB67EH-tfoX-kanR | This study |
| CAS147 | DS40M4 Δ*luxO::Spec^R^*, pCS19 | This study |
| CAS043 | DS40M4 Δ*luxO::Spec^R^*, pMMB67EH-tfoX-kanR | This study |
| CAS211 | DS40M4 Δ*luxR* Δ*luxB::TM^R^* | This study |
| CAS212 | DS40M4 Δ*luxR* Δ*qstR::TM^R^* | This study |
| CAS096 | DS40M4 Δ*luxR::Spec^R^* | This study |
| CAS105 | DS40M4 Δ*luxR::Spec^R^*, pCS19 | This study |
| CAS184 | DS40M4 Δ*luxR::Spec^R^*, pCS32 | This study |
| CAS095 | DS40M4 Δ*luxR::Spec^R^*, pMMB67EH-tfoX-kanR | This study |
| CAS186 | DS40M4 Δ*luxR::Spec^R^*, Δ*luxB::luxR:: TM^R^* | This study |
| CAS188 | DS40M4 Δ*luxR::Spec^R^*, Δ*luxB::luxR:: TM^R^*, pCS19 | This study |
| CAS178 | DS40M4 Δ*luxR::Spec^R^*, Δ*luxB::luxR:: TM^R^*, pMMB67EH-tfoX-kanR | This study |
| CAS181 | DS40M4 Δ*qstR::TM^R^*, pMMB67EH-tfoX-kanR | This study |
| CAS104 | DS40M4, pCS19 | This study |
| CAS213 | DS40M4, pCS32 | This study |
| CAS166 | DS40M4, pMMB67EH-kanR | This study |
| CAS034 | DS40M4, pMMB67EH-tfoX-kanR | This study |
| HY01 | wild-type | (5) |
| CAS106 | HY01, pCS19 | This study |
| CAS209 | HY01, pCS32 | This study |
| CAS168 | HY01, pMMB67EH-kanR | This study |
| CAS071 | HY01, pMMB67EH-tfoX-kanR | This study |
| NBRC 15631 | wild-type (CAIM 519, ATCC 25920) | (6) |
| CAS174 | NBRC 15631 Δ*luxB::TM^R^* | This study |
| CAS142 | NBRC 15631 Δ*luxO::Spec^R^* | This study |
| CAS148 | NBRC 15631 Δ*luxO::Spec^R^*, pCS19 | This study |
| CAS045 | NBRC 15631 Δ*luxO::Spec^R^*, pMMB67EH-tfoX-kanR | This study |
| CAS075 | NBRC 15631 Δ*luxR::Spec^R^* | This study |
| CAS103 | NBRC 15631 Δ*luxR::Spec^R^*, pCS19 | This study |
| CAS217 | NBRC 15631 Δ*luxR::Spec^R^*, pCS32 | This study |
| CAS044 | NBRC 15631 Δ*luxR::Spec^R^*, pMMB67EH-tfoX-kanR | This study |
| CAS102 | NBRC 15631, pCS19 | This study |
| CAS210 | NBRC 15631, pCS32 | This study |
| CAS035 | NBRC 15631, pMMB67EH-tfoX-kanR | This study |
| CAS169 | NBRC15631, pMMB67EH-kanR | This study |
| CAS254 | DS40M4 Δ*luxS* Δ*cqsA,*  *pCS19* | This study |
| TL184 | BB120 Δ*luxS* Δ*cqsA* | (7) |
| TL16 | BB120 Δ*luxS* Δ*luxM* | (7) |
| TL185 | BB120 Δ*cqsA* Δ*luxM* | (7) |
| JMH363 | BB120 *ΔluxM* | (8) |
| TL189 | BB120 *ΔluxM, ΔluxS, ΔcqsA* | (8) |
| CAS270 | BB120 *ΔluxM, ΔluxS, ΔcqsA, pCS19* | This study |
| ***V. cholerae* strains** |  |  |
| E7946 | wild-type 01 El Tor | (9) |
| SAD793 | E7946 Sm^R^ Δ*hapR::Spec^R^*, pMMB67EH-tfox-kanR | This study |
| CAS214 | E7946, pCS32 | This study |
| VC20 | E7946, pMMB67EH-tfoX-kanR | This study |
| CAS172 | E7946 Sm^R^ Δ*hapR::Spec^R^*, pMMB67EH-tfoX-kanR | This study |
| CAS177  VC21 | E7946, pMMB67EH-kanR  E7946 Sm^R^ Δ*luxO::Spec^R^,* pMMB67EH-tfox-kanR | This study  This study |
| ***V. natriegens strains*** |  |  |
| ATCC 14048 | wild-type (NBRC 15631) | (10) |
| CAS215 | wild-type (NBRC 15636), pCS32 | This study |
| CAS039 | Δ*dns::Spec^R^*, pMMB67EH-tfoX-carbR | (10) |
| CAS001 | ATCC 14048, pMMB67EH-tfoX-kanR | This study |
| CAS036 | ATCC 14048, pMMB67EH-carbR | (10) |
| ***V. vulnificus strains*** |  |  |
| ATCC 27562 | wild-type | ATCC |
| CAS-vv013 | ATCC 27562, pMMB67EH-tfoX-kanR | This study |
| CAS-vv015 | ATCC 27562 Δ*smcR::Spec^R^*, pMMB67EH-tfoX-kanR | This study |
| CAS-vv022 | ATCC 27562, pCS32 | This study |
| ***V. parahaemolyticus strains*** |  |  |
| RIMD2210633 | wild-type | ATCC |
| CAS-V01 | RIMD2210633, pMMB67EH-tfoX-kanR | This study |
| CAS-V03 | RIMD2210633 Δ*opaR::Spec^R^*, pMMB67EH-tfoX-kanR | This study |
| CAS-V16 | RIMD2210633, pCS32 | This study |
| ***E. coli* strains** |  |  |
| S17-1λpir | Wild-type, mating strain | (11) |
