# Supplementary material for: Diversity in Natural Transformation Frequencies and Regulation across Vibrio Species
Source: mBio. 2019 Dec 17;10(6):e02788-19. doi: 10.1128/mBio.02788-19 (PMC6918086; doi:10.1128/mBio.02788-19)
Supplement: TABLE S3 [file mBio.02788-19-st003.docx]

**Table S3.** Oligos used in this study.

| Primer Name | primer sequence | Description |
| --- | --- | --- |
| **Primers for mutant constructs** | | |
| lab179 | attccggggatccgtcgac | Ampflify Middle AbR (SpecR, TmR) F |
| lab180 | tgtaggctggagctgcttc | Ampflify Middle AbR (SpecR, TmR) R |
| CAS0001 | gctccgtcagaccactggcaacca | ΔLuxR BB120 F1 |
| CAS0002 | gtcgacggatccccggaatgtccatatttctttttccttgccatttgagttg | ΔLuxR BB120 R1 |
| CAS0003 | gaagcagctccagcctacactatgcatctacaaccgtgaacatcactaaaaaataattag | ΔLuxR BB120 F1 |
| CAS0004 | cgcatctccagttgttcgtcgtct | ΔLuxR BB120 R2 |
| CAS0069 | gtaccaccaatgccagaatcagtgc | NBRC 15361 ΔLuxR F1 |
| CAS0084 | ccacgcagctccttgtccgaatcg | DS40M4 ΔLuxR F1 |
| CAS0070 | gtcgacggatccccggaataggtctctttgcaattgagtccat | NBRC 15361, DS40M4 ΔLuxR R1 |
| CAS0071 | gaagcagctccagcctacaatctacaaccgtgaacatcactaa | NBRC 15361, DS40M4 ΔLuxR F2 |
| CAS0072 | cgactacgcacacgcatctccag | NBRC 15361,DS40M4 ΔLuxR R2 |
| CAS0074 | gtccgtcgctctaccaactgagc | NBRC 15361, DS40M4 ΔLuxO F1 |
| CAS0075 | gtcgacggatccccggaattaccattagtagataacgagac | NBRC 15361, DS40M4 ΔLuxO R1 |
| CAS0076 | gaagcagctccagcctacagtatgaatacggacgtattaaatcagc | NBRC 15361, DS40M4 ΔLuxO F2 |
| CAS0077 | gcttgttcaactaggtagccaccaga | NBRC 15631 ΔLuxO R2 |
| CAS0079 | gtgcttctggcgtgctgtcacg | DS40M4 ΔLuxO R2 |
| CAS0101 | cgtgcttgttcacggctcaagc | HY01 ΔLuxR F1 |
| CAS0102 | gtcgacggatccccggaatctttgcaattgagtccataatcc | HY01 ΔLuxR R1 |
| CAS0103 | gaagcagctccagcctacagatatgctatgcatctacaaccgt | HY01 ΔLuxR F2 |
| CAS0104 | gctcctgcagcagaagcggctc | HY01 ΔLuxR R2 |
| CAS0107 | cgtctcaggcaacgcagacagtg | ATCC 27562 ΔSmcR F1 |
| CAS0108 | gtcgacggatccccggaattgagtccataggttgtttccttacc | ATCC 27562 ΔSmcR R1 |
| CAS0109 | gaagcagctccagcctacagaacacgaatagcaccagtaacctc | ATCC 27562 ΔSmcR F2 |
| CAS0110 | ggaagctcaagcaacgactagtg | ATCC 27562 ΔSmcR R2 |
| CAS0113 | cgatacctgctagcactgccattg | RIMD2210633 ΔOpaR F1 |
| CAS0114 | gtcgacggatccccggaaaggtctctttgcaattgagtccatatcc | RIMD2210633 ΔOpaR R1 |
| CAS0115 | gaagcagctccagcctacacgcgaacactaaagctcagatttg | RIMD2210633 ΔOpaR F2 |
| CAS0116 | gacactggcatgaagatcactccac | RIMD2210633 ΔOpaR R2 |
| CAS0134 | gcagctcgcgatccgaatcatgct | N16961 ΔHapR F1 |
| CAS0135 | ggcttcaaccacacgttcaccat | N16961 ΔHapR R2 |
| CAS0148 | cgtgctcaagtcttcactgatgatg | DS40M4 and NBRC ΔluxB F1 |
| CAS0149 | gtcgacggatccccggaatgatgacttgatcagaagaacgctttga | DS40M4 ΔluxB R1 |
| CAS0150 | gaagcagctccagcctacacactcgtaacgtttaaacgatgctgag | DS40M4 ΔluxB F2 |
| CAS0151 | ggtgaatggccacaaggtacct | DS40M4 and NBRC ΔluxB R2 |
| CAS0206 | gtcgacggatccccggaatgaagaataatccaaatttcatgtctc | R1 delta LuxB NBRC |
| CAS0207 | gaagcagctccagcctacagtcaaataccactcgtaacgtttaaac | F2 delta LuxB NBRC |
| BBC1264 | in ad list | F1 Δdns V. nat |
| BBC1267 | in ad list | R2 Δdns V. nat |
| CAS0247 | ctaggtagatactgctcttctggagag | F1 to delete QstR in DS40M4 |
| CAS0248 | gtcgacggatccccggaatagcatcctcttccatgctgattag | R1 to delete QstR in DS40M4 |
| CAS0249 | gaagcagctccagcctacactgatgtcataaaacaatgatgagcaac | F2 to delete QstR in DS40M4 |
| CAS0250 | caactgaacaagccaacaggaacg | R2 to delete QstR in DS40M4 |
| CAS0252 | gaagcagctccagcctacaataaagtcgacttggtgagtcagtc | F to amplify LuxR to complement into DS4 with homology to Tm cassette |
| CAS0253 | tcgtttaaacgttacgagtgttagtgatgttcacggttgtagatgc | R to amplify LuxR to complement into DS4 with homology to down stream of LuxB |
| CAS0254 | cactcgtaacgtttaaacgatgctg | F2 to amplify down arm of 4 piece SOE product for complementing LuxR or LuxO, by deleting LuxB and replacing with TmR and LuxR or LuxO gene |
| CAS0255 | gaagcagctccagcctacacaacagttggagaaggagatcagtc | F to amplify LuxO to complement into DS4 with homology to Tm cassette |
| CAS0256 | tcgtttaaacgttacgagtgcatacgttttgtttttcgtccttgc | R to amplify LuxO to complement into DS4 with homology to down stream of LuxB |
| CAS0295 | gctaattcagtttaagcggccataggtctctttgcaattgagtccat | R1 to make delta LuxR unmarked in DS40M4 |
| CAS0296 | atggccgcttaaactgaattagcatctacaaccgtgaacatcactaa | F2 to make delta LuxR unmarked in DS40M4 |
| CAS0334 | cacgagcaagatggttgttaagc | F1 V. vulnificus delta pomB(motB) Tm SOE |
| CAS0335 | gtcgacggatccccggaatcatcacatactcccgtgattaatcattg | R1 V. vulnificus delta pomB(motB) Tm SOE |
| CAS0336 | gaagcagctccagcctacagagcagtaattgggtacgtgagttg | F2 V. vulnificus delta pomB(motB) Tm SOE |
| CAS0337 | cacgtcaatgtctggctctttagc | R2 V. vulnificus delta pomB(motB) Tm SOE |
| CAS0339 | gatgttcttcagtgcgacgaaccag | F1 V.para delta pomB(motB) Tm SOE |
| CAS0340 | gtcgacggatccccggaatcatcacaaatctccgcgattactc | R1 V.para delta pomB(motB) Tm SOE |
| CAS0341 | gaagcagctccagcctacagttattcaataacaaagcgcgtc | F2 V.para delta pomB(motB) Tm SOE |
| CAS0342 | gatcgctacatctacatcatcagtca | R2 V.para delta pomB(motB) Tm SOE |
| **Detection primers for mutant constructs** | | |
| CAS0083 | gaagcagctccagcctaca | F detect for all AbR Cassettes |
| CAS0073 | gtgatgcagaagatatcgcac | R detect for NBRC ΔLuxR |
| CAS0078 | tgtcgatggcaatcgctagttcac | R detect for NBRC ΔLuxO |
| CAS0080 | cgtagtcacggagtcattggcttc | R detect for DS40M4 ΔLuxO |
| CAS0085 | gaaggctcaatcactgaccttcc | R detect for DS40M4 ΔLuxR |
| CAS0094 | gttcttgttgctatcggtcgtgttcc | R detect for BB120 ΔLuxR |
| CAS0112 | gcagctccagtagctgcacctg | R detect for ATCC 27562 ΔSmcR |
| CAS0118 | cgtcgtcacttcgaagtgaacg | R detect for RIMD2210633 ΔOpaR |
| CAS0220 | gctgagcatcaatcgctcttgac | DS40M4 ΔluxB detect |
| CAS0251 | ggttcatcacctacaagctcacgac | detect primer delta QstR in DS4 |
| CAS0338 | gagcacttgttggtaatattgatggc | R detect vulnificus delta pomB(motB) Tm SOE |
| CAS0343 | gttgctctcagaggtcacttcaatg | R detect V.para delta pomB(motB) Tm SOE |
| CAS0152 | atggccgcttaaactgaattagc | MASC-PRC Forward detect |
| **Primers for plasmids** | | |
| CAS0125 | agcttggctgttttggcgga | pMMB tfoX F |
| CAS0126 | ggcctatggagctgtgcggc | pMMB tfoX R |
| CAS0127 | actgagcgctgccgcacagctccataggcccttatgaagtccatactttttcactga | PluxC-GFP + homology to pMMB, F |
| CAS0129 | tcttctctcatccgccaaaacagccaagcttcagttgtacagttcatccatgc | PluxC-GFP + homology to pMMB, R |
| CAS0289 | caggatcccgggaggaggtaggcgcgtgaaaaaatcggcttatgcgag | F to amplify QstR DS40M4 insert with homology to pMMB-tfox, includes RBS |
| CAS0290 | tccgccaaaacagccaagctttatgacatcaggttttggtcag | R to amplify QstR DS40M4 insert with homology to pMMB-tfox, |
| CAS0293 | cctcctcccgggatcctgtgtgaaattgaggtcgactctagaggatccttaac | R to amplify the pmmb-tfox backbone to insert QstR |
| CAS0294 | agcttggctgttttggcggatg | F to amplify the pmmb-tfox backbone to insert QstR |
