# Supplementary material for: Diversity in Natural Transformation Frequencies and Regulation across Vibrio Species
Source: mBio. 2019 Dec 17;10(6):e02788-19. doi: 10.1128/mBio.02788-19 (PMC6918086; doi:10.1128/mBio.02788-19)
Supplement: TEXT S1 [file mBio.02788-19-s0001.docx]

**References**

1. Bassler BL, Greenberg EP, Stevens AM. 1997. Cross-species induction of luminescence in the quorum-sensing bacterium Vibrio harveyi. J Bacteriol 179:4043-5.

2. Freeman JA, Bassler BL. 1999. A genetic analysis of the function of LuxO, a two-component response regulator involved in quorum sensing in Vibrio harveyi. Mol Microbiol 31:665-77.

3. Pompeani AJ, Irgon JJ, Berger MF, Bulyk ML, Wingreen NS, Bassler BL. 2008. The Vibrio harveyi master quorum-sensing regulator, LuxR, a TetR-type protein is both an activator and a repressor: DNA recognition and binding specificity at target promoters. Mol Microbiol 70:76-88.

4. Dias GM, Thompson CC, Fishman B, Naka H, Haygood MG, Crosa JH, Thompson FL. 2012. Genome sequence of the marine bacterium Vibrio campbellii DS40M4, isolated from open ocean water. J Bacteriol 194:904.

5. Rattanama P, Srinitiwarawong K, Thompson JR, Pomwised R, Supamattaya K, Vuddhakul V. 2009. Shrimp pathogenicity, hemolysis, and the presence of hemolysin and TTSS genes in Vibrio harveyi isolated from Thailand. Dis Aquat Organ 86:113-22.

6. Baumann P, Baumann L, Reichelt JL. 1973. Taxonomy of marine bacteria: Beneckea parahaemolytica and Beneckea alginolytica. J Bacteriol 113:1144-55.

7. Long T, Tu KC, Wang Y, Mehta P, Ong NP, Bassler BL, Wingreen NS. 2009. Quantifying the integration of quorum-sensing signals with single-cell resolution. PLoS Biol 7:e68.

8. Waters CM, Bassler BL. 2006. The Vibrio harveyi quorum-sensing system uses shared regulatory components to discriminate between multiple autoinducers. Genes Dev 20:2754-67.

9. Miller VL, DiRita VJ, Mekalanos JJ. 1989. Identification of toxS, a regulatory gene whose product enhances toxR-mediated activation of the cholera toxin promoter. J Bacteriol 171:1288-93.

10. Dalia TN, Hayes CA, Stolyar S, Marx CJ, McKinlay JB, Dalia AB. 2017. Multiplex Genome Editing by Natural Transformation (MuGENT) for Synthetic Biology in Vibrio natriegens. ACS Synth Biol 6:1650-1655.

11. de Lorenzo V, Timmis KN. 1994. Analysis and construction of stable phenotypes in gram-negative bacteria with Tn5- and Tn10-derived minitransposons. Methods Enzymol 235:386-405.

12. Furste JP, Pansegrau W, Frank R, Blocker H, Scholz P, Bagdasarian M, Lanka E. 1986. Molecular cloning of the plasmid RP4 primase region in a multi-host-range tacP expression vector. Gene 48:119-31.
